# Spontaneous partial resolution of autoimmune-mediated brain MRI abnormalities before immunotherapy in anti-metabotropic glutamate receptor 5 encephalitis: a case report

**DOI:** 10.3389/fimmu.2025.1568005

**Published:** 2025-05-30

**Authors:** Jingjiong Chen, Xin Tang, Linran Wang, Dandan Guo, Li Cao, Kaili Lu

**Affiliations:** Department of Neurology, Shanghai Jiao Tong University School of Medicine Affiliated Sixth People’s Hospital, Shanghai Neurological Rare Disease Biobank and Precision Diagnostic Technical Service Platform, Shanghai, China

**Keywords:** autoimmune encephalitis, metabotropic glutamate receptor 5, cerebellar dentate nucleus lesions, spontaneous partial regression, case report

## Abstract

Anti-metabotropic glutamate receptor 5 (mGluR5) encephalitis is a rare autoimmune disorder characterized by varied neurological symptoms, often associated with limbic syndromes. We report the case of a 57-year-old female who presented with obvious ataxia, and then cognitive impairment and behavioral changes. MRI revealed cerebellar dentate nucleus and basal ganglia lesions with enhancement, which spontaneously regressed on follow-up imaging—a rare phenomenon in mGluR5 encephalitis. Diagnosis was confirmed by the presence of serum mGluR5 antibodies. Extensive evaluations excluded underlying malignancies. Immunotherapy, including corticosteroids and intravenous immunoglobulin, led to significant clinical and radiological improvement. This case highlights the unique cerebellar dentate nucleus and basal ganglia involvement and spontaneous partial reduction of lesions in mGluR5 encephalitis, expanding the clinical spectrum of this condition. Early recognition and immunotherapy are crucial for favorable outcomes. Further studies are needed to elucidate the mechanisms behind spontaneous partial lesion regression in autoimmune encephalitis.

## Introduction

Anti-metabotropic glutamate receptor 5 (mGluR5) encephalitis is a rare form of autoimmune encephalitis mediated by antibodies targeting mGluR5. First identified by Lancaster et al. in 2011, this condition is often associated with paraneoplastic syndromes, particularly Hodgkin lymphoma, and presents with symptoms such as cognitive impairment, behavioral changes, ataxia, and seizures (1)​. While the limbic system is commonly involved, extralimbic regions, including the basal ganglia, may also be affected (2). Despite growing recognition, the disease remains underdiagnosed due to its rarity and overlapping features with other neurological conditions. This case highlights obvious engagement with cerebellar dentate nucleus abnormalities. Additionally, unlike typical cases which are often linked to persistent or progressive MRI abnormalities, our patient demonstrated spontaneous partial regression of cerebellar dentate nucleus and basal ganglia lesions, a phenomenon seldom reported in anti-mGluR5 encephalitis​. Such findings broaden the clinical and radiological spectrum of the disease. Timely diagnosis using antibody assays and MRI, followed by immunotherapy, is critical in managing this potentially reversible condition. This case underscores the need for heightened awareness among clinicians and adds to the evolving understanding of mGluR5-related encephalitis.

## Case presentation

**Figure 1:**
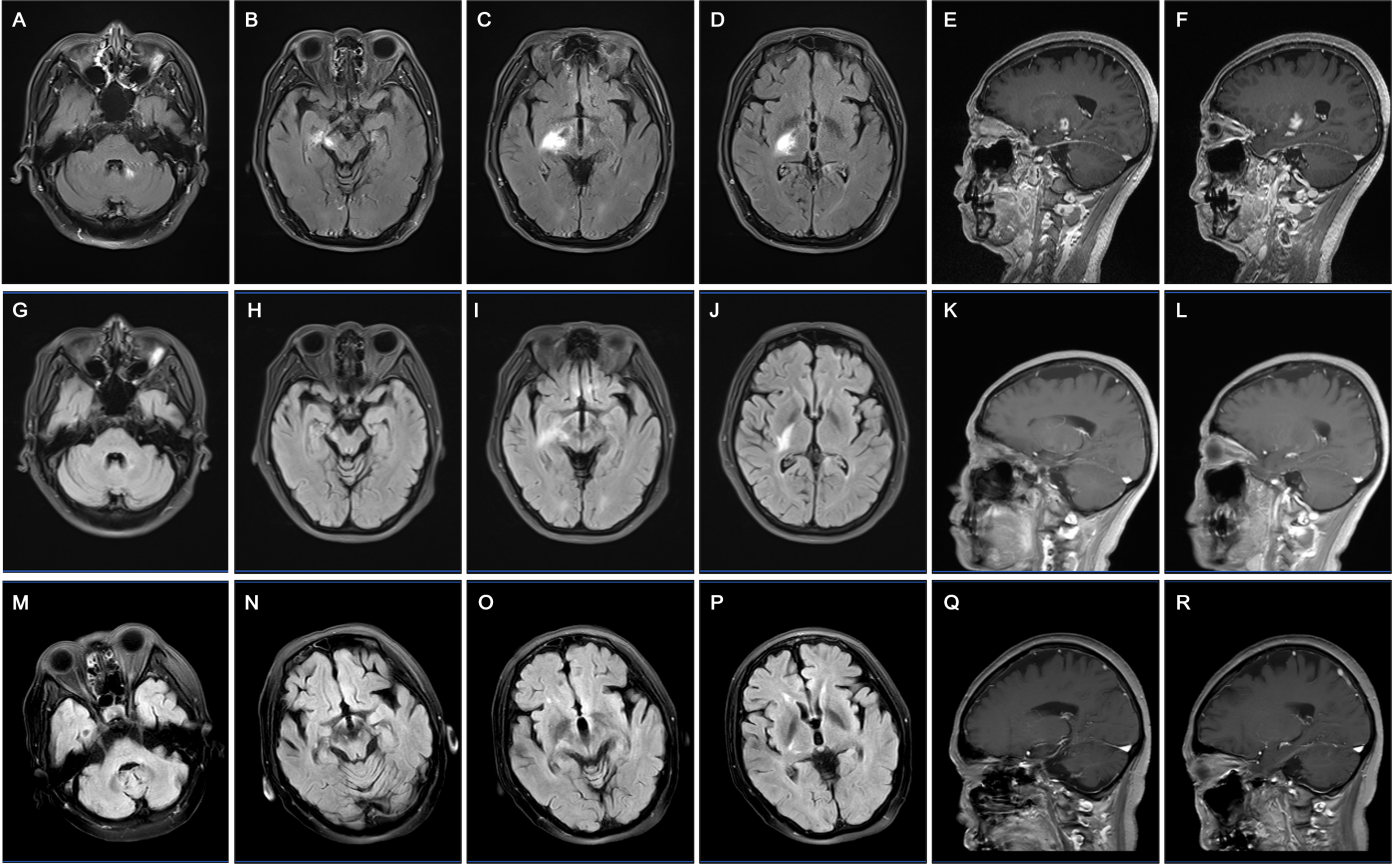
Longitudinal MRI findings in a patient with anti-mGluR5 encephalitis. **(A–F)** Axial and sagittal FLAIR and contrast-enhanced T1-weighted MRI scans in July 2024 reveal hyperintense lesions in the cerebellar dentate nucleus **(A)**, cerebral peduncle in the mesencephalon and partially mesial temporal area **(B)**, right thalamus and posterior limb of the internal capsule **(C, D)**, with pronounced ring and lobulated-enhancing lesions on contrast images **(E, F)**. However, the patient refused further management. **(G–L)** MRI scans in August 2024 show partial reduction of hyperintense lesions in the above regions. Minimal residual enhancement remains **(K, L)**, indicating partial resolution of inflammation. Then the patient received immunotherapy, including intravenous immunoglobulins and methylprednisolone. **(M–R)** MRI scans in January 2025 demonstrate significant resolution of hyperintensities in the cerebellar dentate nucleus **(M)**, cerebral peduncle in the mesencephalon and partially mesial temporal area **(N)**, right thalamus and posterior limb of the internal capsule **(O, P)** without enhancement **(Q, R)**, corresponding with clinical improvement.

A 57-year-old female began in February 2024 with unsteadiness, which were initially ignored. However, by July 2024, her condition had worsened significantly and she presented to a local hospital after experiencing multiple falls. Brain MRI conducted showed abnormal signal with enhancement in cerebellar dentate nucleus, cerebral peduncle in the mesencephalon, partially mesial temporal area, thalamus and posterior limb of the internal capsule, suggesting possible inflammation (**Figure 1**). Initially, the patient’s family refused hospital admission for further treatment. However, due to the patient’s increasing behavioral issues at home, including frequent arguments and irritability, she was referred to our facility for further management in August. Upon admission, her vital signs were stable, and she demonstrated mild cognitive impairment (MMSE: 25/30, MoCA: 14/30). Neurological examination revealed normal cranial nerve function and normal muscle strength (5/5) and tone in all limbs. Deep tendon reflexes were also normal, but both Babinski signs were positive. Coordination testing showed normal finger-nose and Romberg’s test, but difficulty with heel-to-shin maneuvers. And her gait was significantly affected, and she was unable to walk in a straight line. Sensory function was intact, with no abnormalities noted in pain, vibration, or position sensations. The patient’s recent medical history showed no fever, insomnia, or recent vaccinations, and her bowel and urinary function were normal. However, she had experienced a 10 kg weight loss over the past 3 months, accompanied by a noticeable decline in appetite. Unexpectedly, MRI showed a spontaneous partial reduction in cerebellar dentate nucleus, cerebral peduncle in the mesencephalon, partially mesial temporal area, thalamus and posterior limb of the internal capsule region compared to previous scan, while patchy enhancement persisted in the right basal ganglia region (**Figure 1**). Additional diagnostic work-up included EEG, which revealed mild abnormalities with low amplitude α rhythm, scattered low-amplitude θ activity, and occasional sharp waves. Tests for metabolic encephalopathies were negative: complete blood cell count, calcium, magnesium, phosphorus, liver function tests, erythrocyte sedimentation rate, antinuclear antibody, C-reactive protein, thyroid-stimulating hormone, antithyroglobulin, antithyroperoxidase antibodies, cortisol, vitamin B12, and laboratory tests for toxicology. The cerebrospinal fluid (CSF) analysis showed normal pressure and white blood cell count (2 × 10^6/L), along with the mild elevation in protein (0.50 g/L). Importantly, serum mGluR5 antibodies were positive (1:32) on cell-based assay, confirming the diagnosis of autoimmune encephalitis, although CSF testing was negative. Identical oligoclonal bands were found in both serum and CSF. Serum and CSF testing for commercially available neural and non-neural autoantibodies was negative on cell based assay and immunohistochemistry, including against Yo, Hu, Tr, amphiphysin, GAD65(glutamate decarboxylase 65), mGluR1(metabotropic glutamate receptors 1), N-Methyl-D-aspartate (NMDA) receptor, AMPA (α-amino-3-hydroxy-5-methyl-4-isoxazolepropionic acid) receptor, DPPX(dipeptidyl-peptidase-like protein), LGI1 (leucine-rich glioma-inactivated protein 1), GABA _B_R(γ-amino butyric acid type B receptor) and KLHL11. Further evaluation showed the absence of infectious or malignant causes. A PET-CT scan showed a slightly lower-density lesion in the right basal ganglia but no increased glucose metabolism, supporting the diagnosis of encephalitis. The clinical presentation, combined with imaging findings, suggested autoimmune encephalitis, specifically mGluR5 receptor encephalitis. Then the patient was started on immunotherapy, including a 5-day course of intravenous immunoglobulins (IVIG, 0.4g/kg/day) and methylprednisolone (500mg daily for 5 consecutive days), followed by oral prednisone. After treatment, the patient showed significant improvements with modified Rankin score 1, including better appetite, enhanced memory function, and marked improvement in ataxia. In January 2025, the patient was followed up with brain MRI imaging showing further improvement (**Figure 1**). Clinically, the patient had no signs of relapse, and there was no evidence of any tumors.

## Discussion

Anti-mGluR5 encephalitis, a rare and underdiagnosed autoimmune condition, primarily manifests with limbic involvement, often associated with cognitive and behavioral symptoms​​ (3–6). However, our case broadens the clinical spectrum to cerebellar ataxia, involving with basal ganglia, thalamus, brainstem and cerebellum. Such extralimbic presentations have been sporadically noted in previous reports but remain poorly characterized (3, 7, 8)​​. The basal ganglia involvement and noticeable decline in appetite observed in this patient aligns with highly expressed mGluR5 receptors in the basal ganglia motor circuit and gastric vagal afferents (9)​​. Though autoimmune cerebellar ataxia is more common in mGluR1 encephalitis (10), a 29-year-old man with bilateral horizontal nystagmus, ataxia after severe acute respiratory syndrome coronavirus 2 (SARS-CoV-2) vaccination, was reported with mGluR5 antibodies in the cerebrospinal fluid and serum, with abnormal signals in the splenium of the corpus callosum rather than cerebellum (7). In addition, mGluR1 and mGluR5 sharing 85% amino acid sequence homology are both included in group I metabotropic glutamate receptors (1, 9), which may explain the observed cerebellar lesions in our case. These findings suggest that anti-mGluR5 encephalitis might not be as exclusively limbic as initially thought, warranting further exploration into its diverse phenotypic presentations.

The presence of mGluR5 antibodies in the serum confirmed the diagnosis, consistent with reports emphasizing the diagnostic value of cell-based assays. Identical oligoclonal bands in serum and CSF further supported the autoimmune origin. Although limbic system abnormalities dominate typical presentations (4)​, extralimbic lesions, such as those seen in this case, are increasingly reported and suggest a broader disease spectrum. The patient’s spontaneous partial improvement before therapy, observed on follow-up MRI, is a rare phenomenon, which has not been reported and understood in mGluR1 encephalitis​​. However, spontaneous improvement has been sporadically noted in NMDAR and VGKC encephalitis​​ (11, 12). This suggests that a functional rather than a structural neuronal damage underlies the pathogenesis of this disorder (13, 14).

Treatment with corticosteroids and IVIg led to significant clinical improvement, highlighting the reversibility of antibody-mediated damage (3)​​. Immunotherapy, particularly corticosteroids, is well-established as first-line treatment for mGluR5 encephalitis, with the majority of patients achieving partial or complete recovery (2)​. Long-term follow-up, however, is essential, as relapses or delayed tumor associations have been reported in approximately 30% of cases​​ (15, 16). Notably, our patient showed no evidence of malignancy on PET-CT or subsequent surveillance, further broadening the phenotype of non-paraneoplastic mGluR5 encephalitis.

This case underscores several critical points: the importance of recognizing atypical radiological findings in anti-mGluR5 encephalitis, the utility of antibody testing in atypical neurological syndromes, and the potential for spontaneous partial regression. Future research should aim to clarify the immunopathogenic mechanisms underlying these spontaneous changes and explore tailored therapeutic strategies to optimize outcomes for this rare disease.

## Conclusion

In conclusion, this case highlights the importance of recognizing the diverse clinical and radiological manifestations of anti-mGluR5 encephalitis. The patient’s atypical presentation, with basal ganglia and cerebellar involvement, challenges the traditional understanding of this disease, which is often associated with limbic system lesions. The spontaneous partial regression of lesions observed on follow-up MRI underscores the dynamic nature of the disease and the potential for lesion resolution even without aggressive intervention. Early diagnosis through mGluR5 antibody detection and neuroimaging is crucial for timely treatment, and immunotherapy, including corticosteroids and intravenous immunoglobulin, remains effective in improving clinical outcomes. This case adds to the growing body of knowledge on the broader spectrum of mGluR5 encephalitis and emphasizes the need for continued research into the underlying mechanisms of lesion regression and long-term management strategies for this rare and challenging condition.

## Data Availability

The datasets presented in this study can be found in online repositories. The names of the repository/repositories and accession number(s) can be found in the article/supplementary material.
